# Larval honey bees infected with *Nosema ceranae* have increased vitellogenin titers as young adults

**DOI:** 10.1038/s41598-017-14702-4

**Published:** 2017-10-26

**Authors:** Lee R. BenVau, James C. Nieh

**Affiliations:** Section of Ecology, Behavior, and Evolution, Division of Biological Sciences, University of California, San Diego, 9500 Gilman Drive, MC0116, La Jolla, CA 92093 USA

## Abstract

*Nosema ceranae* is a pervasive and widespread honey bee pathogen that is associated with colony declines and has recently been shown to infect larval honey bees. In adult bees, *Nosema* infection is known to alter levels of a key protein, vitellogenin (Vg), which is necessary for egg-laying in queens, brood food production in workers, and proper immune function in all female bees. We therefore tested the effects of larval worker infection on hemolymph Vg titers. In 1-day old adult workers that were infected as larvae with 10,000 (10 K) or 40,000 (40 K) live *N. ceranae* spores/bee, Vg titers were significantly elevated by + 83% and + 73%, respectively, as compared to controls. At 7 days of adult age, Vg remained significantly elevated (+ 68%) in 10 K treated workers as compared to control workers. *Nosema* infection decreased total hemolymph protein titers in 1 and 7-day old adult bees (−50% in the 10 K and 40 K treated bees). Bees infected as larvae also had a more queen-like sting morphology. They developed slightly but significantly fewer barbs on their stings (−7% in the 40K-treated bees). Higher Vg levels are associated with younger bees. Thus, elevated Vg levels could delay normal age polyethism and disrupt colony balance.

## Introduction

Since 2006, beekeepers have reported unusually high winter colony losses, 22–30% and higher in the USA^1,2^. Some European countries have also reported similar losses^2–4^. These losses have been caused by a combination of factors such as pesticides, parasites, pathogens, and impaired nutrition that act synergistically to decrease honey bee health^5^. The spread of the microsporidian pathogen, *Nosema ceranae*, from its original host, the Asian honey bee (*Apis cerana*), to infect the European honey bee, *A. mellifera* has compounded this problem of poor honey bee health^6–8^. *N. ceranae* infection decreases bee health by weakening the immune system and causing energetic stress^9,10^. *N. ceranae* is associated with colony declines^1,6,11^.

*N. ceranae* infection has multiple effects on adult bees^10,12–15^. Previously, it was suggested that *Nosema* only infects adult honey bees^16,17^, although recent work demonstrated that larvae can also be infected^18^. Larvae fed live *N. ceranae* spores showed fully formed spores within their midgut cells as prepupae and were significantly more infected than controls, based upon midgut spore counts, upon adult death^18^. However, little was known about the effects of larval *N. ceranae* infection, perhaps because this pathogen was believed to only infect adult honey bees^16,17^. In general, studying the effects of larval pathogens is important because a variety of larval diseases can affect development and adult morphology. For example, deformed wing virus (DWV) infects larvae and leads to crippled wings and bloated abdomens in adults^19^. DWV is not known to interact synergistically with *N. ceranae* infection^20^, and to date, no studies have investigated the effects of *N. ceranae* infection alone on honey bee morphology.

*Nosema* infection can also alter normal bee polyethism, the progression of tasks that a bee engages in as it ages^15^. Healthy adults typically begin foraging when they are 22 days old^21^. *Nosema ceranae* infection can prematurely accelerate this polyethism, causing infected nurse bees to adopt the behaviors of foragers at an earlier age^22^. Goblirsch *et al*.^23^ showed that nurse-aged bees infected with *N. ceranae* were twice as likely to engage in precocious foraging as non-infected controls. Recently, Lecocq *et al*.^15^ found that newly emerged adult bees, which were infected with *N. ceranae* spores and observed for 14 d, exhibited traits of older bees: increased walking and decreased attraction to queen mandibular pheromone. These forward shifts in age polyethism may be adaptive for the colony and limit the spread of infection^24^ because foragers have a higher probability of dying outside the nest than nurse bees. Likewise, honey bees treated with drugs or otherwise impaired tend to leave the colony^25^. Even a slight shift in age polyethism could be beneficial for the colony by increasing the time that infected bees spend outside the nest. For example, *N. ceranae* may be transmitted via food exchange^26^. A colony may limit pathogen spread if infected nurse bees refrain from feeding larvae, moving instead towards the next steps, producing wax and building combs^21^.

Acceleration of age polyethism should alter multiple physiological traits that are normally associated with bee aging. For example, vitellogenin (Vg) is a glycolipoprotein that is a precursor to the major yolk protein, vitellin, and is found in females of all oviparous species^27,28^. In insects, it is synthesized in fat body cells and released into the hemolymph^29^. Vg is necessary for egg-laying and is used by nurse bees to produce the protein-rich glandular secretions that are fed to brood^30^. Vg is also a potential endocrine factor, and helps to regulate important aspects of the honey bee life cycle including age polyethism^31,32^ and lifespan^32,33^. Newly emerged workers have low Vg levels that reach a peak at around 4 days^34^. As these nurse bees age, their Vg stores decrease and they gradually transition to becoming foragers^30^. In bees infected as adults, *N. ceranae* can decrease adult Vg levels^9^, resulting in Vg levels more similar to those found in older bees such as foragers. Antunez *et al*.^9^ showed that there is a significant decrease in Vg gene expression in 14-day old adults infected with *N. ceranae* at 7 days of age. Thus, young adults infected with *N. ceranae* develop Vg levels that appear age-accelerated.

However, nothing was known about how *N. ceranae* infection contracted by honey bee larvae alters subsequent adult Vg levels. We therefore examined how *N. ceranae* infection of larvae affects adult hemolymph Vg and total hemolymph protein levels. We hypothesized that larval *Nosema* infection would result in young bees with lower hemolymph Vg levels. We focused on hemolymph because Vg occurs in multiple tissues, but Fluri *et al*.^35^ showed that hemolymph Vg titer is well correlated with honey bee age polyethism. In addition, *N. ceranae* infection causes energetic stress that can induce adult bees to consume more food^10,12^. In our *in vitro* rearing assay, we sometimes observed control-treatment workers that appeared larger and more queen-like as compared to newly emerged adult bees naturally reared in colonies. We therefore examined the effects of larval infection on an indicator of queen-like morphology, the number of barbs on an adult sting^36^.

## Results

### No effect of treatment upon short-term survival

We reared our larvae to adulthood using standard *in vitro* techniques^18,37^. Our 4-day-old larvae (*n* = 253) were fed zero, 10 K (10,000), or 40 K (40,000) freshly prepared *N. ceranae* spores as a single dose. Fresh spores were extracted and purified on the day of use. Overall, 81% of larvae survived to adulthood, and there was no significant effect of treatment on the number of bees that survived to adulthood (*χ* ^2^ = 3.42, 2 d.f., *P* = 0.18). There was also no significant effect of treatment upon adult survival within 7 days (Proportional Hazards survival model: Wald *χ* ^2^ = 0.95, 2 d.f., *P* = 0.62 and no significant colony effect, Wald *χ* ^2^ = 2.97, 3 d.f., *P* = 0.40).

### Larvae fed live spores were significantly infected as compared to controls

**Figure 1:**
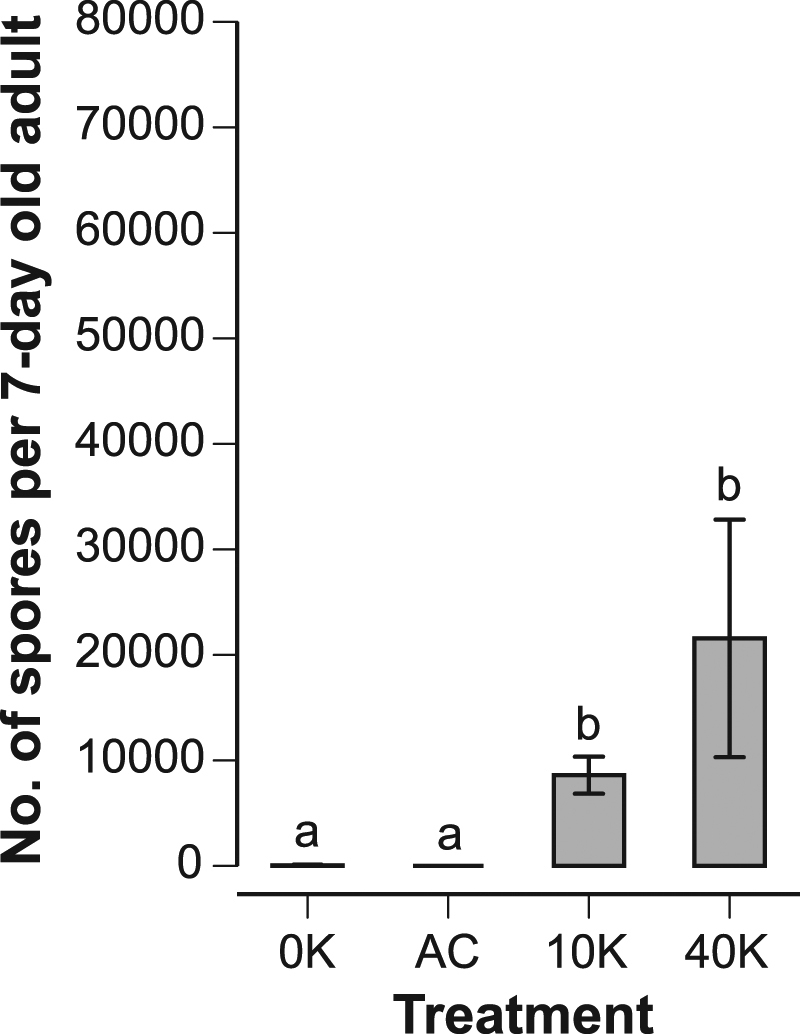
Effect of spore treatments on subsequent spore loads in 7-day old adult bees. Bees were fed spores (0 K = no spores, AC = heat-killed spores, 10 K = 10,000 spores, 40 K = 40,000 spores) as 4-day old larvae. Different letters indicate significant differences (*P* ≤ 0.001). Standard error bars are shown (*N*_bees 0K_ = 43, N _bees AC_ = 27, *N*_bees 10K_ = 41, and *N*_bees 40K_ = 32).

In the larval experiment, larvae fed 10 K or 40 K of live *Nosema* spores were significantly more heavily infected than bees fed either control (no spores or autoclaved spores: Steel-Dwass comparisons, Z ≥ 3.67, *P* ≤ 0.001, Fig. 1). There was no significant difference between the spore counts of bees given the 10 K or 40 K treatments (Steel-Dwass, *Z* = 1.48, *P* = 0.45). Likewise, there was no significant difference between the two control treatments (0 K vs. AC: Steel-Dwass, *Z* = 0.76, *P* = 0.87).

### Larvae fed live spores had elevated Vg titers as adults

**Figure 2:**
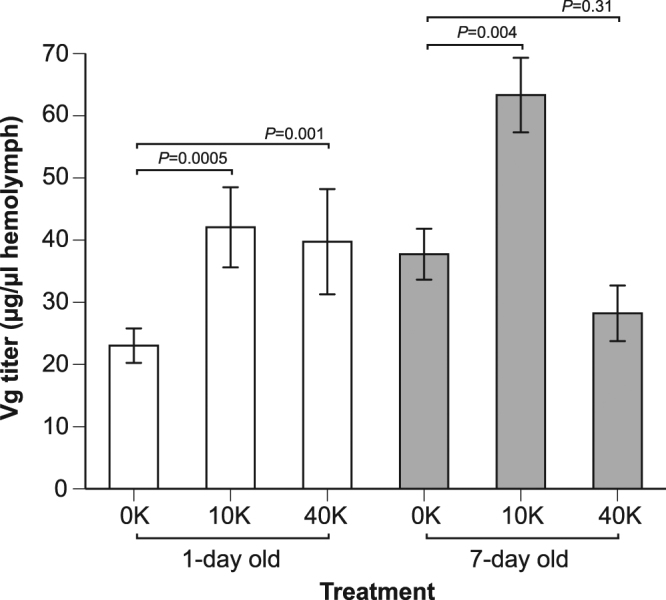
Effect of larval exposure to *Nosema* upon subsequent hemolymph Vg levels in 1-day and 7-day old adult bees. Contrast test results and standard error bars are shown (*N*_1-d bees 0K_ = 39, *N*_1-d bees 10K_ = 35, *N*_1-d bees 40K_ = 31, *N*_7-d bees 0K_ = 22, *N*_7-d bees 10K_ = 18, and *N*_7-d bees 40K_ = 22).

There was a significant effect of treatment on adult Vg titers (*F*_2,158_ = 8.50, *P* = 0.0003, *n* = 167 bees, Fig. 2). Colony accounted for 26% of model variance. There was no significant effect of age (*F*_1,146_ = 0.29, *P* = 0.59), but there was a significant interaction of age*treatment (*F*_2,158_ = 6.28, *P* = 0.002, Fig. 2). In 1-day old bees, 10 K and 40 K treatments elevated Vg titers by 83% (LS means contrast test: *F*_1,158_ = 8.16, *P* = 0.005) and 73% (contrast test: *F*_1,160_ = 10.94, *P* = 0.001), respectively, compared to the control treatment. In 7-day old bees, the 10 K treatment increased Vg titers by 68% (contrast test: *F*_1,158_ = 8.51, *P* = 0.004) compared to the control. The 40 K treatment did not have different Vg levels from the control (contrast test: *F*_1,158_ = 1.06, *P* = 0.31, Fig. 2). Thus, larval infection with *Nosema* (both doses) strongly increased Vg titers in 1-day old adults. At 7 days of age, this effect was confined to the 10 K dose.

### Larvae fed live spores had decreased hemolymph protein titers as adults

**Figure 3:**
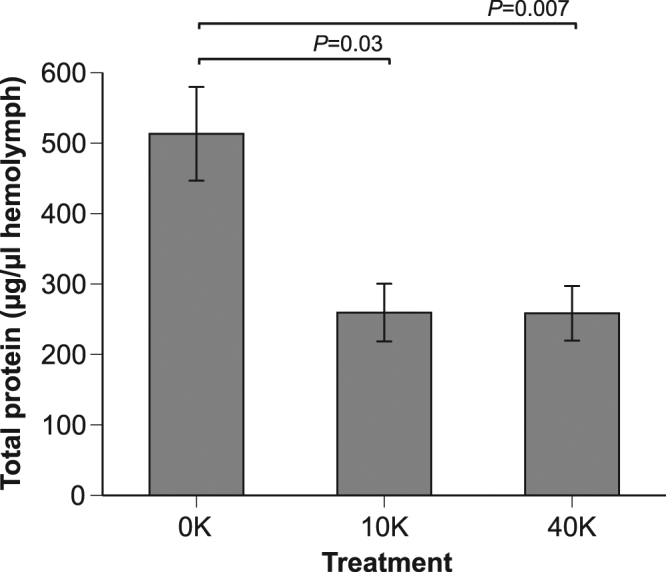
Effect of larval exposure to *Nosema* upon subsequent total adult hemolymph protein levels in 1-day and 7-day old adult bees. The data from 1-day and 7-day adults were pooled because there was no significant effect of age (see Results). Contrast test results and standard error bars are shown (*N*_bees 0K_ = 28, *N*_bees 10K_ = 16, and *N*_bees 40K_ = 25).

There was a significant effect of treatment on the total protein titers (*F*_2,60_ = 4.28, *P* = 0.02, *n* = 69 bees, Fig. 3). There was no significant effect of age (*F*_1,4_ = 3.67, *P* = 0.13) and no significant interaction of treatment*age (*F*_2,59_ = 0.10, *P* = 0.91). Colony accounted for 20% of model variance. The 10 K (*F*_1,65_ = 4.93, *P* = 0.03) and 40 K treatments (*F*_1,56_ = 7.87, *P* = 0.007) both resulted in significantly lower (50% decrease) protein titers than the control treatment (Fig. 2).

### Larvae fed live spores had fewer barbs on their stings as adults

**Figure 4:**
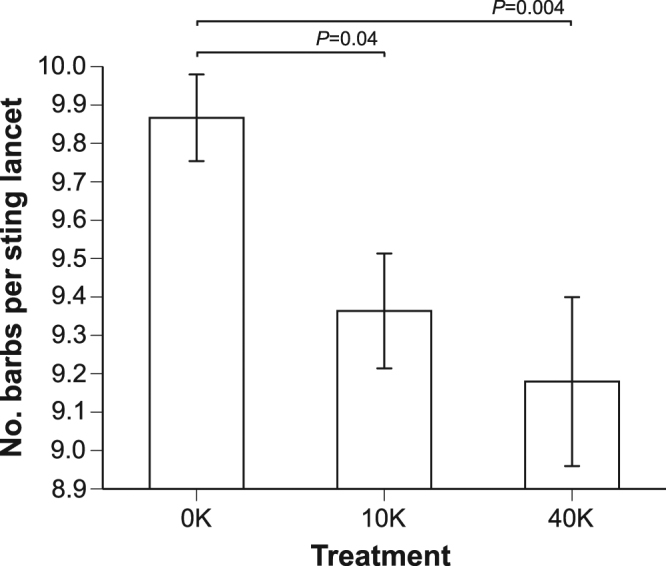
Effect of larval exposure to *Nosema* upon subsequent adult sting morphology. The data from 1-day and 7-day adults were pooled because the number of barbs is already fixed upon adult emergence. Workers typically have 8–11 barbs/lancet and queens have 2–5 barbs/lancet^36^. Contrast test results and standard error bars are shown (*N*_bees 0K_ = 45, *N*_bees 10K_ = 33, and *N*_bees 40K_ = 39).

There was a significant effect of treatment (*F*_2,113_ = 4.82, *P* = 0.0098, *n* = 117, colony accounted for 1% of model variance). Both 10 K (*F*_1,114_ = 4.48, *P* = 0.036) and 40 K bees (*F*_1,110_ = 8.91, *P* = 0.004) had slightly but significantly fewer barbs than control bees (Fig. 4). Thus, larval *N. ceranae* infection caused the developed adults to have slightly fewer barbs (7% fewer) on their stings as compared to controls.

## Discussion

*Nosema* infection contracted by adults can significantly decrease their Vg levels^23^. Given the recent finding that larvae can also be infected with *N. ceranae* ^18^, we tested the effects of larval *Nosema* infection upon Vg levels in young adults. Surprisingly, bees infected as larvae showed the opposite effect: they had unexpectedly high levels of Vg as young adults. In 1-day old bees infected as larvae, 10 K and 40 K treatments elevated Vg titers by 83% and 73%, respectively, compared to the control treatment. At 7 days of age, this effect was confined to the 10 K dose, which resulted in a significant Vg elevation (68%) compared to 7-day old controls. Larval *Nosema* infection decreased total hemolymph protein titers by 50% in 7-d adults as compared to controls. Finally, bees infected as larvae had a more queen-like morphology; they developed slightly fewer barbs on their stings (1-day 40k bees had 7% fewer barbs than control bees).

### Larval infection levels

Bees infected as larvae have detectable but low spore counts as young adults^18^. These spore counts are lower than those typically found in bees infected as adults. Although *N. ceranae* fed to larvae evidently survives through bee metamorphosis and gut remodeling^18^, these processes may result in diminished spore proliferation. Larval exposure may also lead to infection latency or spore dormancy. The relatively low spore counts in 7-day old bees are perhaps not surprising given that *Nosema* spore counts increase significantly as bees age, particularly in foragers that are 22–45 days of age^38^. Full infection of the adult ventriculus occurs 10–12 days after inoculation, but, at 7 d, spore counts have reached about 80% of this level^39^. Using older bees would likely have resulted in higher spore counts. However, as bees age, their levels of Vg markedly decline^34^. Vg levels are highest in nurse bees at around 4 d^34^. At 7 d, Vg levels have declined >30% from this peak and at 15 d they have decreased >90%^34^. We therefore chose 7 d, a time point at which control bees should still have high Vg levels and be infected for a sufficient duration to produce detectable *Nosema* spore counts. As expected^18^, the spore counts in our larval-infected bees were low (Fig. 1), but they were the result of an active infection, not due to the retention of fed spores because larvae fed heat-killed spores (AC) did not have any spores in their midguts as adults (Fig. 1).

### Vitellogenin titers

Larvae fed *Nosema* had unexpectedly higher levels of Vg as young adults. It is possible that our *in vitro* rearing of honey bee larvae, which we used to control for multiple factors such as the hygienic removal of diseased larvae by nurse bees, may have influenced Vg levels. *In vitro* rearing, which provides larvae with a diet rich in royal jelly, could stimulate Vg production. Specifically, levels of Vg in 1-day old control bees were higher than those reported in naturally reared bees^40^. Amdam *et al*.^40^ showed that naturally reared and uninfected 2-day and 8-day old adult honey bees (*A. mellifera*) had Vg titers of approximately 5 µg/µl and 45 µg/µl, respectively. In our *in vitro* reared control bees, the 1-day old bees had higher Vg titers (22 µg/µl) while our 7-day old control bees were like the naturally reared 8-day old bees (38 µg/µl, Fig. 2). The potential Vg-elevating effects of *in vitro* rearing had therefore largely disappeared by 7 days of adult age. Nonetheless, at 7-days of age, *Nosema* treated bees still had higher Vg levels (10 K treatment) than controls (Fig. 2). Thus, although *in vitro* rearing may have elevated overall Vg levels, *Nosema*-treated bees still had higher Vg levels than control bees of the same age.

At 7 d of adult age, bees that were fed 10 K, but not 40 K, of spores as larvae had significantly elevated Vg levels, but not significantly different levels of infection (Fig. 1). Eiri *et al*.^18^ applied the same treatments and suggested that the 40 K dose activated larval immunity because 40 K bees were significantly less infected than 10 K bees upon adult death (a much longer time span than 7 d). If true, such immune activation likely occurs at an early age and may impose a cost that is reflected in the lower Vg levels measured at 7 d in 40 K bees. This possibility should be explored in greater detail.

Goblirsch *et al*.^23^ demonstrated that 8-day old adult bees had decreased Vg gene expression in comparison with controls when adult bees were inoculated (10 K live spores fed to bees within 24 h of emergence). In contrast, we found that 4-d old larvae inoculated with 10 K live spores had significantly elevated Vg levels at 7 d (Fig. 2). This difference likely arose because larval inoculation altered the developmental trajectory of Vg expression in a fundamentally different way from adult inoculation. Given the tight linkage between juvenile hormone (JH) levels and Vg expression^41^, it would useful for a future study to compare the effects of larval and adult *Nosema* inoculation on JH levels^23^.

### Total protein titers

*Nosema* infection evidently harms bee nutrition. Infection results in lower protein levels and consequently reduces the size of the hypopharyngeal glands, which produce brood food secretions^22,42,43^. If fewer nutrients are absorbed by infected midguts, then total hemolymph protein titer could decrease. This could explain why the 40 K larval-infected bees had lower levels of total protein than control bees (Fig. 3). Cage rearing of bees may also have influenced these protein levels. Lass and Crailsheim^44^ showed that caged bees have significantly lower protein synthesis rates than bees of the same age in a normal colony. Nonetheless, in the 40 K larval treatment group, total protein levels were lower than in the control group (Fig. 3).

### Sting morphology

The effects of our treatments on barb counts were slight (5–7%), but nonetheless significant given the relatively low level of variance within each treatment (Fig. 4). An alternative interpretation of our sting morphology results is that workers were not more queen-like, but simply had deformed stings with fewer barbs (Fig. 3). This is possible, but the sting shafts and the shapes of the barbs did not show any malformation. Moreover, the adults were also free of noticeable morphological defects. Why, therefore, was sting morphology more queen-like? If larvae infected with *N. ceranae* exhibited the elevated hunger levels known to occur in infected adults^10^, they may have consumed more of the *in vitro* diet and therefore more royal jelly, leading to a more queen-like morphology. Whether this would occur in natural rearing is unknown, but this is a testable hypothesis in colonies with persistently high levels of *Nosema* infection.

### Implications of larval infection

Bees infected as adults tend to have much higher levels of *Nosema* infection^38^ than bees infected as larvae^18^. Our results suggest that infection level may not be the only measure of harm. Infected larvae suffered, as adults, from decreased total hemolymph protein levels and temporarily elevated Vg levels. Interestingly, the effects of infection were markedly different depending upon whether bees are infected as adults^23,45^ or larvae (Figs 2 and 3).

Bees infected as adults exhibited higher juvenile hormone titers, and correspondingly lower Vg gene expression^23^. Both of these traits characterize older bees that engage in precocious foraging^23^ or other tasks performed by older workers^15^. This accelerated age polyethism may be adaptive since infected bees could transition to work that takes them outside of the hive and reduces their contact with nestmates^25^. Larval infection seemed to have the reverse effect. The elevated Vg titers in newly emerged adults suggests that these bees were “decelerated.” We predict such bees would spend more time as nurses and more easily spread their pathogen to larvae or adults inside the hive. However, bees infected as larvae had low spore counts (Fig. 1) compared to bees infected as adults^39^. Thus, nurse bees infected as newly emerged adults may pose more of a threat to larvae than nurse bees infected as larvae. Nonetheless, this scenario raises the intriguing possibility that *Nosema* could have some control over its host, perhaps changing host behavior to increase parasite fitness. Future studies examining larval infection in natural colonies, the spore doses that larvae could naturally receive from nurses, the effects of larval infection on juvenile hormone titers and the age of first foraging, and how *Nosema* spreads within a colony will be necessary to test these hypotheses.

## Methods

### Colonies

We used bees from four different healthy colonies that were determined to be free of *Nosema* infection and *Varroa* parasitism using standard techniques^3,46^. These colonies were kept at the UCSD Biological Field Station apiary.

### Spore preparation

We used freshly prepared *N. ceranae* spores maintained at room temperature and provided to bees within less than 24 hrs after purification. To generate spore stock, we fed stock bees with approximately 100,000 spores/bee. These bees were only fed sucrose solution, not pollen, to ensure that their gut contents primarily consisted of spores. To obtain spores, we follow previously described methods^18^. We measured spore concentrations with a hemocytometer in a compound microscope (Zeiss Axioskop), making two independent measures of each sample and recording the average spore count^47^. We confirmed that our spores were *N. ceranae* by sequencing them (primer pairs NoscRNAPol-F2 and NoscRNAPol-R2 from)^48^ and comparing our sequences with GenBank data.

### Larval infection experiment

Bees from four healthy colonies that were free of *Nosema* infection were raised *in vitro* based upon the methods of Aupinel *et al*.^37^ and modified as described in Eiri *et al*.^18^. Four-day-old larvae were treated with freshly harvested 10,000 (10 K) *N. ceranae* spores, 40,000 (40 K) spores, or 0 (0 K) spores added to the basic larval diet near their heads in 10 μL of fluid of sterile ddH_2_0. Bees were frozen at −70 °C at two time points: upon adult emergence or 7 days after emergence (chosen because 80% of the ventricular cells have been found to show *Nosema* parasites 7 days post-infection^49^).

The number of spores that larvae are naturally fed remains unknown and deserves further study. However, feeding larvae 10 K or 40 K of live spores can yield significantly infected pre-pupae and adult bees^18^. The realistic spore doses to which even adult bees are exposed remains unclear. Huang and Solter^50^ found that an oral cavity rinse of 14–15 d old worker bees yielded 2010 ± 1133 spores/bee (mean ± 1 standard deviation) based upon qPCR results. However, Fries *et al*.^51^ noted that doses of 10,000–33,000 spores per bee are often fed to adult bees in *Nosema* experiments and 10X higher doses are routinely used to ensure infection. Determining realistic oral exposure to *Nosema* spores for adults and larvae is, nonetheless, important and should be a research priority. In our case, we note that larvae are naturally fed over multiple days by nurse bees and could be incrementally exposed to spores.

To determine if our larval *Nosema* treatments infected bees, we counted spores (see above) in the midguts of treated bees at 7 days of adult age. To confirm that the spores counted in the bees’ midguts resulted from infection, and were not the original spores fed to the bees, we conducted a separate experiment^18^. Each larva was individually fed 40,000 heat-killed spores (AC) in 2 μl of 2.0 M sucrose solution. These spores were harvested as described above, placed inside an Eppendorf microcentrifuge tube, autoclaved for 20 min at 121 °C, and then recounted in a hemocytometer to confirm the spore concentration.

Newly emerged adults were placed in cages, each with half of a queen pheromone lure to simulate a queen-right condition (PseudoQueen, Contech Inc.). After 7 d, the surviving bees were frozen and prepared for hemolymph extraction as described below. We chose 7 d because it is within the window of peak hemolymph Vg in 5–10 day old bees^34^ and is also the length of time that Antunez *et al*.^9^ used for their *Nosema*-infected bees. Although Vg in normal one-day old bees is typically too low to detect^34^, we measured adult Vg levels upon emergence to test for a potential effect of *N. ceranae* infection. We also measured the infection levels of adult bees upon death by carefully dissecting out intact midguts into distilled water and counting spores in a hemocytometer (see above).

### Vitellogenin and protein quantification

Bees were stored at −70 °C prior to use. Bees were thawed for 10 min at room temperature and their mouthparts were glued shut with cyanoacrylate adhesive^52^. We then used dissecting scissors to sever the legs close to the body and placed the bee in a microcentrifuge tube with a membrane-less insert (Costar Spin-X #9301) that allowed hemolymph to flow freely through. The bees were then centrifuged for 1 min at 1000 rpm in an Eppendorf 5415 D centrifuge (modified methods of Mayack *et al*.^53^). To reduce hemolymph clotting, which could limit extraction, bees were prepared in small groups of typically five individuals to minimize the time between severing the legs and centrifugation. We were able to extract 2 μl of hemolymph from most bees per treatment group, similar to the 2 µl/bee obtained by Mayack *et al*.^53^. Although this was a small volume, it was more than sufficient to examine its color and transparency. We did not use hemolymph samples that were contaminated by ruptured abdomens (cloudy or yellow in color) or bees in which the hemolymph extraction did not yield at least 1 μl (<10% of samples). To ensure uniformity, our analysis focuses on clear 2 µl samples of hemolymph.

Hemolymph samples were then frozen (−70 °C) for later purification or immediately purified with the SDS-PAGE Sample Prep Kit (Pierce Biotechnology, Rockford, IL, USA # 89888) according to the manufacturer’s recommendations. The purification yielded 50 μl of protein solution. We used 25 μl to measure Vg levels and 12.5 μl to assay total protein levels, reserving the remaining 12.5 µl as a backup.

To isolate and quantify Vg, we used sodium dodecyl sulfate polyacrylamide gel electrophoresis (SDS-PAGE) and the bicinchoninic acid (BCA) assay^54^. SDS-PAGE is widely used to separate honey bee Vg from other hemolymph proteins^29,55–57^. The BCA assay is used to quantify Vg and other hemolymph proteins after separation with SDS-PAGE in honey bees and other insects^29^.

Hemolymph samples were run on 7% acrylamide gels with a 4% stacking gel at 200 V at 21 °C for approximately 1 hr, or until the protein bands were sufficiently resolved^58^. The gels were run on a Mini-PROTEAN Tetra Cell (Bio-Rad #165–8000), and we used a Thermo Scientific Spectra Multicolor High Range Protein Ladder (#26625) that included a 180 kDa protein marker. *Apis mellifera* Vg is a 180 kDa protein^29^. Gel bands corresponding to 180 kDa were excised into 120 μl of buffer (50 mM Tris-HCl, 150 mM NaCl, and 0.1 mM EDTA; pH 7.5), homogenized with a motorized Kontes pestle for 1 min, and the protein eluted for 24 hours. The samples were then centrifuged for 10 min at 10,000 rpm, and 100 μl of the supernatant was collected to measure protein content.

To measure total hemolymph protein, we followed the same procedure, but did not run our samples through the SDS-PAGE gel. We placed 12.5 μl of the hemolymph in 87.5 μl of the elution buffer for a total volume of 100 μl (matching the Vg samples) and ran this mixture through the BCA assay. We followed the manufacturer recommended protocol for the BCA assay (Thermo Scientific Kit # 23225) and used a spectrophotometer (Pharmacia Biotech Ultrospec 2000, model # 80-2106-00) to visualize protein concentrations.

### Sting morphology

In a preliminary experiment, we noticed that some larvae infected with *Nosema* appeared larger and therefore more queen-like as compared to naturally-reared workers. To quantify this, we counted the number of barbs per sting lancet, since this is a standard measure that yields, clear, discrete values^36^. Queens have fewer barbs per sting lancet (2–5 barbs/lancet) compared to workers (8–11 bars/lancet)^36^. We used forceps to pull out both lancets of the sting from an adult bee, randomly selected one lancet for measurement, placed this on a slide, covered it with a drop of water and a coverslip, and viewed it under 100x magnification (Zeiss Axioskop compound microscope) to count the number of sting barbs/lancet.

### Statistics

To determine if our treatments resulted in infected adults, we used non-parametric Wilcoxon/Kruskal-Wallis tests because the spore count data were not normally distributed. To perform all pairwise comparisons, we used Steel-Dwass tests for non-parametric multiple comparisons that are corrected to reduce the possibility of Type I error. We used Microsoft Excel V14.6.1 to run a *χ* ^2^ test on the number of bees that survived to adulthood. To analyze adult survival, we ran a Proportional Hazards model with colony as a fixed factor. For all tests, we used JMP V13.0 statistical software.

We used Analysis of Variance (ANOVA, REML algorithm) to test for treatment effects (fixed) on Vg titers, protein titers, and barb number. These data met normal assumptions as determined through inspection of the residuals^59^. In each model, colony was a random effect. To compare individual treatments, we used limited contrast tests based upon graphical data inspection. We report mean ± 1 standard error.

### Data availability

All data used in our analyses is available as a Supplemental Dataset included with this paper.

## Electronic supplementary material

Dataset 1
